# The cerebro-cerebellar system: Integrative roles in motor control, cognition, and neuropsychiatric disorders

**DOI:** 10.1016/j.ibneur.2026.03.001

**Published:** 2026-03-03

**Authors:** Mst. Mohona Khatun, Mohammad Shahangir Biswas, Munna Kumar Podder, Rubait Hasan, Mst. Ayesha Siddika

**Affiliations:** a Department of Biochemistry and Biotechnology, Khwaja Yunus Ali University, Enayetpur, Chowhali, Sirajganj, Bangladesh; b Department of Biochemistry and Biotechnology, University of Science & Technology Chittagong (USTC), Foy's Lake, Zakir Hossain Road, Chittagong 4202, Bangladesh; c Department of Public Health, Daffodil International University, Dhaka 1216, Bangladesh

**Keywords:** Cerebellum, Cerebral Cortex, Cerebro-cerebellar Loops, Neuroplasticity, Long-Term Depression, Schizophrenia, Autism Spectrum Disorder, Ataxia, Neuroimaging, Neuromodulation

## Abstract

The cerebro-cerebellar system, a network of bidirectional loops between the cerebellum and cerebral cortex, is crucial for coordinating motor control, cognition, and emotion. Moving beyond its traditional role in motor coordination, contemporary research underscores the cerebellum's significant involvement in higher-order functions, including executive control, language, and social cognition. This review synthesizes current anatomical, functional, and clinical evidence to delineate the cerebellum’s role in neuropsychiatric and neurodegenerative disorders such as schizophrenia, autism spectrum disorder (ASD), and ataxia. Unlike previous broad reviews, this article provides a structured framework that links cerebellar functional topography with disease-specific pathophysiological mechanisms. We particularly highlight the cellular substrates of cerebellar plasticity, such as Purkinje cell long-term depression (LTD). Our synthesis reveals that prior literature often lacks mechanistic depth and fails to integrate findings across structural, functional, and clinical domains. We conclude by proposing future research directions involving advanced neuroimaging, artificial intelligence, and targeted neuromodulation, thereby clarifying the cerebellum’s integrative roles and underscoring its translational potential for novel therapeutic strategies.

## 1 Introduction

The cerebro-cerebellar system is a highly interconnected network that is at the center of both motor and cognitive processes. By using feedback and prediction mechanisms, this system maximizes a variety of processes through dynamic interactions between the cerebellum and the cerebral cortex (Bansal et al., 2024). Starting in the cerebral cortex, the cerebro-cerebellar circuitry passes through the mossy fibre system to the pons and finally to the cerebellar cortex. The passing of modified information from the cerebellum before being sent on to the deep cerebellar nuclei and then back to the cerebral cortex relies on this route through the thalamus (Koziol and Leonard, 2014). The olivary system, which links the cerebellum with the inferior olive, (Wang et al., 2022) adds yet another layer of sophistication to this system. The cerebellum is widely recognized for its responsibility in prediction calculations—internal forward models. By using such models, which support movement and thought reactions and hence are imperative to motor control, motor practice, and mind, the cerebellum can foretell sensory repercussions of movement instructions. The cerebellar output being predictive as affirmed by anatomical and physiological information, which reflects that they are predictive of cerebellar input (Tanaka et al., 2020, Sultan et al., 2012). Functional neuroimaging studies like diffusion-weighted MRI and fMRI suggest functional compartmentalization in the cerebro-cerebellar system. Cognitive functions involve mostly the posterior cerebellum; motor functions involve other segments of the cerebellum, hence pointing to their functional specialization (Salmi et al., 2010). The many connections between the cerebellum and other regions of the brain underscore even more its dual implication in motor and cognitive function (Leiner et al., 1991). The cerebellum communicates with the cerebral cortex and basal ganglia in an integrated cerebello-basal ganglia-thalamo-cortical system. The system mediates a broad array of motor and cognitive functions, thus emphasizing the function of the cerebellum beyond traditional motor control (Caligiore et al., 2017). Precision and spatial coordination of inputs characterize the operation of the system, thus justifying the role performed by the cerebellum in adaptive prediction and error-based learning (Watson and Apps, 2021, Sokolov et al., 2017). It is required to recognize the relationship between cerebellum and cerebrum since recent research has revealed the role of cerebellum in social cognitive processes like "mind" and "body" reading. Social reasoning and judgment rely on the functional association of the cerebellum with cerebrum, and these have a significant effect. Cerebellar activity reflects certain mentalizing and mirroring processes about corresponding networks in cerebrum (Van Overwalle et al., 2015, Van Overwalle et al., 2020). likewise the cerebellum helps regulate an array of cortical functions via domain-general adaptation. They have less regular networks compared to the cerebral cortex, which is appropriate for their function in adaptive regulation for cognition and motor functions (Marek et al., 2018). The cerebellar contribution in higher cognitive functions like attention, language, and emotional regulation emphasizes even more its interaction with the cerebrum (Zhang et al., 2023, Wright et al., 2016). The understanding of motor and cognitive function dynamics requires an integrated system made up of cerebrum, basal ganglia, and cerebellum. The interconnectivities within such a system define learning and the mechanism of control, highlighting the cerebellum's role in both motor and non-motor processes (Caligiore et al., 2017). Cerebellar involvement in cognitive neural networks is particularly through cerebral-cerebellar network loops and greatly involves cognitive neurological functions. Evolutionarily, the ontogeny of the human cerebellum with a surface area of about 80% that of the neocortex indicates that it is at the core of human-specific cognition and activities like language and tool use. Anatomical correlation with the cerebrum indicates the cerebellum's role in the emergence of higher-order cognitive functions (Sereno et al., 2020). The review article focuses on the effective and functional relationship between the cerebellum and cerebrum, with emphasis on their roles in cognitive processes and social cognition. Combining the results of many studies can allow one to fully grasp the relationships among different brain regions. This involves examining the interface of function and architecture, the place of cerebro-cerebellar circuits in cognition, and the implications of these interfaces on understanding brain structure and development. This essay synthesizes the findings in an attempt to delineate how the cerebellum subserves cognitive and social function and thereby highlights the relevance of the study of the cerebrum and cerebellum as a single system. Earlier publications neglected to integrate molecular data or bring to the fore contributions specific to a particular disease, tending instead to inventory cerebellar structure or overarching clinical correlations. This study was spurred by this disparity.

The contributions of the review and the failings of previous works. While numerous reviews have presented the general clinical correlations or the organization of the cerebro-cerebellar system, they tend to provide only descriptive information without an integration of different levels of analysis in a meaningful way. The literature now in press is haunted by the serious lack of paying insufficient attention to specific mechanisms involved in the cerebellar lesion and varied symptom patterns in neuropsychiatric disorders. Furthermore, the findings from cellular plasticity, anatomy, and functional imaging are not sufficiently synthesized in reviews to provide a logical schema that can be used in translational research. To fill these lacunae, this review provides a systematic synthesis that:

1. Explicitly connects disease-specific pathways in disorders like ataxia, schizophrenia, and ASD to cerebellar functional sub-specialization.
2. Incorporates coverage of plasticity biological mechanisms (e.g., Purkinje cell LTD), clinical phenomenology, and anatomical connectivity.
3. Highlights major knowledge gaps and suggests specific future directions to fill them, including the use of personalized neuromodulation and artificial intelligence.

## 2 Methodology

Methodology and Sources of Information Major academic databases for examples PubMed/MEDLINE, Scopus, and Google Scholar, were searched in order to give a comprehensive picture of the cerebro-cerebellar system. Peer-reviewed publications, clinical trials, and review papers released between 2000 and 2024 were the main focus of the search. Keywords and Search Terms: The following keywords were used with the Boolean operators (AND, OR) to conduct the literature search:

• "Cerebro-cerebellar pathway."
• "Cerebellar-cortical loops."
• "Cerebellum and executive function."
• "Cerebellum in schizophrenia and ASD."
• "Non-motor functions of cerebellum."
• "Ataxia and cerebellar functional sub-specialization."

Criteria for Study Selection and Inclusion Based on the keywords, the search initially produced about 320 records. 145 papers were selected for full-text review after duplicates were removed and titles and abstracts were screened. Articles that satisfied the following requirements were accepted:

1. Highlight the connections between the cerebrum and cerebellum, either anatomically or functionally.
2. Reviewing the role of the cerebellum in neuropsychiatric or neurodegenerative diseases.
3. Recent developments in cerebellar pathway-related neuroimaging methods.

Omitted studies were those that were not available in English or that only addressed spinal cord injury. Finally, this review synthesized 137 articles.

### 2.1 Anatomy and general overview of the cerebro-cerebellar system

The human brain comprises two primary, distinct but highly interconnected structures: the cerebral cortex (cerebrum) and the cerebellum. The cerebrum is the largest component of the brain, divided into two hemispheres and four distinct lobes—occipital, temporal, parietal, and frontal—which handle higher-order functions such as movement, reasoning, sensory perception, and visual processing (Gurses et al., 2023). Morphologically, the cerebral cortex is characterized by a convoluted layer of grey matter containing gyri (ridges) and sulci (grooves), a structural adaptation that maximizes surface area and neural capacity (Heuer et al., 2023). Within, the cerebrum relies on a complex structural connectome of axonal channels—including association, projection, and commissural fibers—to facilitate integration within and between hemispheres, thereby supporting global network efficiency. ^19–21^

The cerebellum is found in the posterior cranial fossa, located dorsal to the brainstem (Błaszczyk et al., 2024, Roostaei et al., 2014). Physically, it is made up of two lateral hemispheres connected by a median structure known as the vermis. Similar to the cerebrum, the cerebellar surface is extensively folded into folia, which significantly increases neural density within a compact volume (Adekomi, 2017). The vascular supply to the cerebellum is obtained from three primary arteries: the superior cerebellar artery (SCA), the anterior inferior cerebellar artery (AICA), and the posterior inferior cerebellar artery (PICA), assuring the metabolic demands of its high neuronal population are met (Petersen et al., 2019).

The structural connectivity between these two organs is mediated by polysynaptic pathways that form the basis of the cerebro-cerebellar system (Buckner et al., 2011, Singh, 2020). Two principal pathways facilitate this bidirectional communication: the Cortico-Ponto-Cerebellar (CPC) pathway and the Dentate-Rubro-Thalamo-Cortical (DRTC) pathway (Keser et al., 2015, Palesi et al., 2015). These loops are responsible for planning and executing movements as well as supporting higher mental functions.^30,31^In addition, dopaminergic outputs to the superior colliculus (SC), with connections to the basal ganglia, play a role in behavioral control (Melleu and Canteras, 2024, Wedeen et al., 2012).

**Table 1:** Principal Features of the Cerebrum and Cerebellum.

**Features**	Cerebrum	Cerebellum
Location	The largest part of the brain is divided into two hemispheres (Gurses et al., 2023).	Beneath the cerebrum, behind the brainstem in the posterior cranial fossa (Gurses et al., 2023, Roostaei et al., 2014).
Lobes	Frontal, parietal, temporal, and occipital lobes (Gurses et al., 2023).	Two lateral hemispheres connected by the vermis (Gurses et al., 2023, Adekomi, 2017).
Surface Characteristics	Gyri (ridges) and sulci (grooves) (Heuer et al., 2023)	Highly folded surface with folia (Heuer et al., 2023, Adekomi, 2017).
Primary Functions	Sensory perception, cognition, motor control (Zhang et al., 2023, Diedrichsen et al., 2019).	Motor control, cognitive regulation, and emotional processing (Popa and Ebner, 2019).
Key Pathways	Cortico-cortical axonal pathways, structural core (Yeh et al., 2018, Hagmann et al., 2008).	CPC pathway, DRTC pathway, cerebro-olivocerebellar pathway (Keser et al., 2015, Palesi et al., 2015).
Blood Supply	Middle cerebral artery, anterior cerebral artery, and posterior cerebral artery (Gurses et al., 2023).	Superior cerebellar artery, anterior inferior cerebellar artery, posterior inferior cerebellar artery (Błaszczyk et al., 2024).

The cerebrum serves as the main center for higher-order cognitive functions, including perception, language and decision-making (Guell et al., 2018). In the meantime, the cerebellum is now broadly acknowledged for its essential modulatory influence on these same processes (Likova et al., 2021). Additionally, its classic role in motor coordination, the cerebellum refines cognitive and emotional functions through predictive processing and error correction mechanisms (McAfee et al., 2022, Shahshahani et al., 2023). The interplay between these regions supports optimal functioning across motor, cognitive, and affective domains. Table 2 outlines these integrated roles.

**Table 2:** The merged functions of the cerebrum and cerebellum in motor, cognitive, and emotional processes.

**Operational Area**	**Role of Cerebrum**	**Role of Cerebellum**	**Synergistic Interaction**
Motor Regulation & Neuroprosthetics	The planning and initiation of voluntary movements are mediated by the primary motor cortex (M1) as well as the premotor cortex (Abbasi et al., 2024).	The process increases motor commands for accuracy, equilibrium, and temporal coordination; it modulates M1 activity (Abbasi et al., 2024).	The ensuring of smooth execution of movement; cerebellar inhibition prevents motor errors.
Cognitive Operations	The process is characterized by the execution of advanced which encompass decision-making, working memory, and language (McAfee et al., 2019).	The entity modulates neocortical inputs to facilitate working memory and mental imagery; it interacts with the default mode network (DMN) (Popa and Ebner, 2019).	Bidirectional loops enable swift error correction in cognitive functions and linguistic fluency.
Emotional Control	The mechanisms that facilitate the conscious emotional experience are mediated through the prefrontal cortex and the amygdala (Guell et al., 2018).	Regulates emotional states and interpersonal responsiveness (e.g., 'Theory of Mind') (Likova et al., 2021).	The system coordinates emotional reactions with social context through limbic–cerebellar connections.
Sensorimotor Integration	The system analyzes external sensory information to produce motor plans.	Functions as a “Predictive Model” to foresee the sensory outcomes of actions (Tanaka et al., 2020).	The system facilitates online predictive control and effortless adaptation to variations in the environment.
Temporal Alignment	The process begins with a series of tasks.	Coordinates neural oscillations to ensure precise timing of cognitive and motor events (McAfee et al., 2019).	The synchronization of neural activity is essential for performing tasks that necessitate high temporal precision, such as speech and music.

### 2.2 Structural relationship between the cerebrum and cerebellum

The compound and intricate relationship between cerebrum and cerebellum of two-way associations keeps a vast spectrum of the brain activities under control in primates functionally. The two organs suffered qualitative changes to functions with the evolutionary process; the cerebellum is far more important as far as knowledge is concerned currently. The system of cerebello-cerebral is approximately scaled; both halves tend to increase together in size. A good example of a cerebellar area that has evolved more quickly than the cerebellum or cerebrum as a whole is the lateral cerebellar lobules crura I-II (ansiform lobules) (Magielse et al., 2023). There are several pathways, with primarily the cortico-ponto-cerebellar tract and the cerebello-thalamo-cortical circuit linking the cerebellum anatomistically with the cerebrum. The evidence for the intimate inter-connections of the cerebellum with higher-order centres of cognition comes from these frontal-lobe biassed circuits (Jobson et al., 2024). Also, there is a disynaptic circuit reported in which cerebellar output nuclei are connected to the basolateral amygdala via the intralaminar thalamus as an intermediate node (Jung et al., 2022). This is the first mechanistic account of the cerebellar contribution to emotional processing.

The cerebellum and cerebrum are sophisticated interacting components in functions. Various lobules of the cerebellum have functional correspondence to distinct brain areas, based on fMRI studies (Ren et al., 2021). For example, bilateral posterolateral cerebellum has been associated with multilingual language control; left cerebellum with prefrontal, parietal, and subcortical cognitive control regions, and the right cerebellum with largely language control. Furthermore, functional connections between most cortical networks and the ventral attention, motor, and auditory networks of the cerebellum exist. Most prominent is cerebellar-cerebral hyperconnectivity pathology in conditions such as schizophrenia (Kim et al., 2020). The abnormally connected component points to the significance of cerebellar-cerebral communication in neuropsychiatric disease that is primarily targeted to specific cerebellar areas such as Crus I, Crus II, and lobules IX and X. Both structural and functional relationships between various cerebral and cerebellar locations have been shown by recent research. Visual bodily movement can be calculated on a structural loop between left cerebellar lobule Crus I and the right posterior superior temporal sulcus (STS). [47] Also functionally related to the cerebellum are brain areas for psychomotor alertness: the inferior frontal gyrus, caudate, and postcentral gyrus (Zhang et al., 2019). Also linked with numerous cognitive functions is the cerebro-cerebellar connection. Rapid automatic naming, for instance, is connected with the relationship of the left supramarginal gyrus to bilateral cerebellum VI, but reading phonological awareness with the relationship between the left insula and the right cerebellar VI (Ang et al., 2020). Also, control over multilingual language is associated with bilateral posterolateral cerebellum, but is more precisely with the right cerebellum. Advances in technology in neuroimaging and cerebellar atlases continue to sharpen this intricate cerebro-cerebellar interaction (Lyu et al., 2024).

### 2.3 Functional association of the cerebrum and the cerebellum

In addition to motor coordination, the functional collaboration between the cerebrum and cerebellum includes complex cognitive and emotional domains via specific neural circuits.

### 2.4 Motor control

Learning, motor control, and coordination all depend on the cerebellum. To coordinate posture, balance, and dexterity, it translates sensory inputs from the vestibular apparatus, proprioceptors, and vision. The cerebellum maintains smooth and accurate movement by reconciling the sensory information with the motor commands (Zhang et al., 2019). Its function in the storage of dynamic motor frequencies, enabling homogeneous motor kinematics among individuals, has been highlighted in recent publications (Magielse et al., 2023, Jobson et al., 2024). Besides, the cerebellum is involved in motor learning, enabling people to improve skills by practice and feedback—a very important skill for sports and musical instrument playing, for example (Ang et al., 2020).

Cerebellar neuronal circuits, such as the cortico-cerebellar loop and cerebello-spinal pathway, are involved in complex locomotor learning, interlimb coordination, and rapid motor adaptation by error input via climbing fibers (Lyu et al., 2024, Groiss and Ugawa, 2013, Cullen, 2023). Structures such as deep cerebellar nuclei (DCN) and unipolar brush cells (UBCs) augment motor acuity and adaptation learning (Sathyamurthy, 2023, Liu et al., 2024). Cerebellar deficiency results in motor and cognitive dysfunctions, including dystonia, ataxia, chorea, athetosis, cerebellar cognitive affective syndrome (CCAS), and posterior fossa syndrome (PFS), highlighting the role of the cerebellum in motor as well as non-motor functions (Baumann and Jason, 2022, Alahmadi, 2023, Guo et al., 2020, Schäfer et al., 2020).

The cerebrum processes sensory data and produces motor commands to activate and prepare actions, especially in the primary motor cortex and premotor cortex. In order to keep movements rhythmic and precise, neural oscillations of the cerebrum integrate action between motor areas (Chéron et al., 2016). The motor cortex and prefrontal cortex, which are tasked with decision-making and movement modifications, collaborate to improve motor output based on feedback (Buhusi et al., 2018). The cerebrum can also act as a "timing machine," accurately controlling movement length in milliseconds, according to research (Bareš et al., 2019).

Coordination between the cerebellum and cerebrum allows voluntary movements to be planned and performed. For accuracy, balance, and coordination, the cerebellum refines motor commands following movement initiation by the cerebrum (Palesi et al., 2015). Neural oscillations within the cerebellum are synchronized with the forebrain to facilitate learning of skills in motor learning (Halverson et al., 2023). Predictive control is made possible by the two structures' mutual interconnection, enabling the cerebellum to refine and improve actions in real time (Van Overwalle et al., 2019). Making accurate fine motor movements, gait coordination, and posture maintenance all depend on this interconnection (Askoxylakis et al., 2017, Coombes and Misra, 2016).

### 2.5 Cognitive functions

There is increasing evidence for the cerebellar role in higher-order cognitive processes like memory, language, and executive function. For cognitive operations like working memory, speed of processing, and cognitive flexibility, some of these areas, such as Lobule VIIB, Crus I, and Crus II are specialized (van der Heijden and Sillitoe, 2023, Joshua et al., 2022, Hatten, 2020). From functional neuroimaging research, the cerebellum is engaged in verbal working memory and sequence learning tasks (Mastrangelo et al., 2024).

The right hemisphere of the cerebellum is necessary for language control in bilingual subjects, speech regulation, word fluency, and sentence formation (Maldonado and Bernard, 2021, Ashida et al., 2019). Cerebellar cognitive affective syndrome (CCAS) is a neurologic disorder where executive function, language, and emotional regulation are impaired due to damage to the cerebellum (Rudolph et al., 2023). Apart from this, through interaction with prefrontal and parietal regions of the brain, the cerebellum controls cognitive control and emotional processing.

Cerebro-cerebellar loops facilitate cognitive processing by bidirectional communication between the cerebellum and cerebral cortex. These loops are characterized by bidirectional communication, allowing the cerebellum to modify and coordinate cognitive functions, just as it modifies and coordinates motor control (Zhang et al., 2023). Functional MRI studies during working memory tasks, planning, and cognitive flexibility show cerebellar activity, especially in the posterior cerebellum (Clark et al., 2021). The cortico-ponto-cerebellar circuits have been made more differentiated by high-resolution tractography, underscoring their function in cognitive processing. [74] Cognitive impairments, especially executive function, can be induced by disruptions in these circuits, such as cerebellar stroke or frontotemporal dementia (Chen et al., 2020, Tan et al., 2015).

### 2.6 Emotional regulation

The cerebellum's connection with the limbic system and cerebral cortex yields emotional learning, affective state, and mental illness. Neuroimaging and clinical research attest to its cognitive-affective processing, which impinges on autonomic and affective regulation (Clark et al., 2021). Posterior cerebellum is associated with mentalizing, or theory of mind, to understand others' emotions, and with the consolidation of emotional memories, both important for social and emotional learning (Van Overwalle, 2024). Social behavior regulation is linked with dopamine D2 receptors in cerebellar Purkinje cells, suggesting that the cerebellum plays a role in influencing reward-related affective states (Cutando et al., 2022). Its involvement in emotion processing is also seen from the observation that certain areas of the cerebellum become active during emotional experiences and aggressive reactions (Klaus and Schutter, 2021). Cerebellar role in mood control is highlighted by structural and functional dysfunctions of mental illnesses such as major depressive disorder, bipolar illness, and schizophrenia. The cerebrum, which includes the hippocampus, amygdala, and prefrontal cortex, is one of the most significant components of emotional regulation. The cerebellum utilizes cerebro-cerebellar loops to optimize and change such processes for effective emotional responses. Autism, ADHD, and mood disorders are among the neurodevelopmental and psychiatric illnesses that are linked to impairments of these networks (Mastrangelo et al., 2024, Phillips et al., 2015).

### 2.7 Sensorimotor integration

The cerebellum and cerebrum collectively coordinate sensory input and motor output to produce accurate, smooth, and coordinated movement. The cerebellum refines motor commands by pre-programming the outcome of movement and correcting in real time, while the cerebrum deals with external sensory information and produces motor commands (Tanaka et al., 2020). As an internal forward model, cerebrum-cerebellum network anticipates sensory outcomes of motor action and modifies motor output in response. In the case of reaching and grasping movements that involve fine motor coordination, such predictive control is essential (Zahra et al., 2022). Repeated experience is seen to enhance motor predictions by the cerebellum, and that is how motor learning occurs (Calame et al., 2023). Disturbances in cerebello-cerebral circuits can interfere with motor precision and contribute to motor disorders such as ataxia and epilepsy (Chen et al., 2024). By and large, sensorimotor integration is achieved well by interaction between cerebellum and cerebrum in order to allow adaptive motor control and online error correction with volitional movement (Tanaka et al., 2020, Zahra et al., 2022, Calame et al., 2023).

### 2.8 Clinical relevance of cerebro-cerebellar impairment

Cerebrum and cerebellum are the parts of the brain responsible for motor, cognitive, and affective functioning. Parkinson’s, autism, and schizophrenia are some of the neurological and psychiatric illnesses associated with impairment of these systems, requiring special treatment (Miterko et al., 2019). Motor impairments like ataxia and dystonia have a significant correlation with cerebellar impairment. Coordination impairments are the hallmark of ataxia, which is most frequently linked to cerebellar degeneration and can result in cognitive impairment, as demonstrated by cerebellar cognitive affective syndrome (CCAS) (Liu et al., 2025). The intricate relationship between motor and cognitive function in inherited ataxia has been demonstrated by studies that have established strong correlations between the severity of ataxia and cognitive test performance (Khafizova et al., 2024, Shin et al., 2024). The interaction of motor and cognitive impairment in the cerebro-cerebellar model is also evidenced by the fact that disorders such as vertebrobasilar insufficiency can worsen cognitive impairment (Selvadurai et al., 2024). Higher-order cognitive domains like memory, language, and executive function all suffer significantly due to the influence of the cerebellum. Neuropsychiatric illnesses like schizophrenia, ADHD, and ASD are associated with structural and functional impairment of the cerebellum. Evolving evidence links specific symptom clusters to distinct cerebellar sub-regions. For example, in schizophrenia, cognitive deficits are increasingly linked with disrupted connectivity between the right cerebellar Crus I/II and the left medial prefrontal cortex. Equally, social deficits in Autism Spectrum Disorder (ASD) have been correlated with structural abnormalities in cerebellar lobule VII and the vermis, regions critical for social cognition and emotional processing (KaSP, Moberget et al., 2018, D’Mello and Stoodley, 2015). Cognitive impairments involving working memory and attention are part of the cerebellar pathology-related cognitive deficits in schizophrenia (Gill and Sillitoe, 2019, Faris et al., 2024). Cerebellar implication in social behavior and emotion modulation is suggested by the observation that social behavioral impairments in ASD and ADHD arise from anatomical pathology in discrete cerebellar subregions (Dong et al., 2020, Bègue et al., 2023). As illustrated by cerebellar cognitive affective syndrome (CCAS), emotional lability and changes in personality also result from damage to the cerebellum (Palesi et al., 2017). Given its association with the prefrontal cortex as well as with other brain regions, the cerebellum remains a prime candidate for therapeutic applications because of these associations, underpinning the role of the cerebellum in emotional and cognitive processing (Zhang et al., 2023). The cerebellum is susceptible in some instances, since it is among the first brain structures to show signs of illness in conditions like chronic traumatic encephalopathy (CTE). Increased cerebellar activity has been theorized to be a compensatory process to counteract early neurological symptoms in neurodegenerative diseases like Parkinson's disease (PD) and Alzheimer's disease (AD) (Palesi et al., 2017). In motor diseases such as ataxia and dystonia, cerebellar dysfunction is also responsible for the impairment of sensory integration (Liu et al., 2022). There is promise for improving cognitive and motor outcomes by modulating cerebellar function with non-invasive brain stimulation techniques like transcranial magnetic stimulation (TMS) and transcranial direct current stimulation (tDCS). Anodal tDCS over the medial cerebellum, for instance, improves cognitive function, while high-frequency TMS over the lateral cerebellar hemispheres can induce cognitive impairment (Pezzetta et al., 2024). Such observations demonstrate the therapeutic potential of cerebellar manipulation for treating disorders such as schizophrenia and ASD (Lupo et al., 2018). The brain also reorganizes functionally after cerebellar damage, with networks related to motor having greater interhemispheric coupling. This would imply that rehabilitation should be an effort to reorganize patterns of functional activation with focused therapies, such as brain stimulation and physiotherapy (De Vico Fallani et al., 2017). Cerebellar stimulation has been explored as an add-on to medication to treat ataxia and coordination disturbances. TMS and tDCS have both been applied as an adjunct or alternative to pharmacological therapies for the purpose of enhancing residual cerebellar circuit function (Ferrucci et al., 2018). Rehabilitation methods based on neurophysiological strategies such as robotic and visual biofeedback have been reported to decrease symptomatology and enhance coordination in individuals with MS-related cerebellar ataxia (Chasiotis et al., 2023). Motor and cognitive impairments have been found to be decreased by interventions like exercise and cognitive training. Motor-cognitive training, a mix of motor and cognitive tasks, has been found to enhance motor coordination and executive function in older adults (Levin et al., 2017). Patients with cerebral small vascular disease (SVD), where white matter atrophy results in impairments in executive function and motor coordination, benefit the most from these methods (Jokinen et al., 2022).

### 2.9 Neuroplasticity

The capability for neuroplasticity of the cerebellum allows it to remodel circuitry in response to learning, environmental stimulation, or injury. Plasticity underlies motor skill learning and general cognitive processing. Through practice and sensory experience by repetition, movement internal models become fine-tuned such that precision is acquired and movement is automatized. Structural changes, such as increased synaptic density after practice of movement also attest to cerebellar involvement in motor adaptation (Cai et al., 2014). Beyond movement per se, plasticity of the cerebellum underlies integrated functionality with the neocortex such that cognition, attention, working memory, and executive control are possible. Neuroimaging and stimulation research demonstrates that cortically dynamic processing involving the cerebellum facilitates compensation after injury and preserves predictive computations underlying flexibility and planning (Reichert et al., 2017, Gatti et al., 2021, Price and Duman, 2020).

Best described at the cellular level are long-term depression (LTD) and long-term potentiation (LTP) between parallel fibers and Purkinje cells. Thought once to be the dominant system for motor learning, LTD is recognized to act together with LTP to produce a bidirectional and reversible basis for adaptive learning (Geminiani et al., 2024). Synaptic plasticity in other than these types—including between mossy fibers and granule cells, between mossy fibers and Golgi cells, between parallel fibers and interneurons, and between Golgi cells and granule cells—is additional to intrinsic plasticity—excitability changes in neurons not mediated by synapses (Locatelli et al., 2019, Ohtsuki et al., 2020). Different groups of neurons are engaged in these activities. Purkinje cells, which are the sole outflow neurons from the cortex of the cerebellum, add excitatory input from granule cell parallel fibers and modulatory input from interneurons. Granule cells, by their massive numbers (the most abundant neurons in the brain), spread mossy fiber input through parallel fibers to Purkinje cells and inhibitory interneurons. Interneurons from the molecular layer (stellate and basket cells) fine-tune Purkinje activity, and golgi cells regulate firing in granule cells; all these networks exhibit plasticity (Soler-Llavina and Sabatini, 2006). All these systems together enable fine-grained regulatory control over both cognitive and motor activities. Experimental evidence validates behavioral assessments of cerebellar plasticity. Motor learning is spurred by LTD and LTP in Purkinje synapses and abolition in mouse models of ataxia and autism results in motor impairment (Mapelli et al., 2016, Neureither et al., 2017). Cortical plasticity and excitability in motor networks are controlled by cerebellar output in man, demonstrated by behavioral experiments and transcranial brain stimulation (Goldenkoff et al., 2025). Neuroimaging also demonstrates disrupted activation patterns with cerebellar disease and enhanced corticocerebellar connectivity with successful stroke (Guell and Schmahmann, 2020). Dysfunction of plasticity mechanisms is apparent in disease. In progressive ataxia, loss ultimately outpaces compensatory ability, generating long-term defects (Mitoma et al., 2020). Autism has abnormal development of the cerebellum along with decreased Purkinje cells and defects in plasticity at synapses, leading to social and motor impairments (Jaber, 2017). Schizophrenia entails structural and functional defects in the cerebellum as well, though positive cognitive effects from stimulation of the cerebellum continue to be researched, and some symptoms have been shown to improve (Hua et al., 2022). Finally, therapy methods increasingly try to involve cerebellar plasticity. Therapies such as transcranial direct current stimulation (tDCS) and vigorous rehabilitation have shown efficacy in enhancing motor and cognitive measures by creating adaptive plasticity (Liebrand et al., 2020). Future directions include refining neuromodulation methodologies, tailoring therapy to patient profiles, introducing behavioral interventions, and employing sophisticated imaging to define compensatory plasticity in cerebellar and extracerebellar networks (Jun et al., 2024). The principal forms of plasticity, their respective cell types, and the implicated synapses are found in Table 3.

**Table 3:** Categories of Cerebellar Plasticity & their Physiological Implications.

**Plasticity Types**	**Key synapses involved**	**Functional Roles**
LTD/LTP	Purkinje cell-parallel fiber.	Memory, motor learning, bidirectional adaptation (Geminiani et al., 2024).
	Mossy fiber-Golgi, mossy fiber-granule cell.	Input gain control, information filtering, timing (Locatelli et al., 2019).
	Parallel fiber-molecular layer interneuron	Network modulation, inhibition (Soler-Llavina and Sabatini, 2006).
Intrinsic plasticity	Granule, Golgi cells, purkinje	Modulates neuronal excitability (Ohtsuki et al., 2020).
GABAergic plasticity	Golgi cell-granule cell	Balances excitation or inhibition (Locatelli et al., 2019).

### 2.10 Current Trends and Future Directions

Recent studies have reaffirmed the relationship between the cerebellum and brain through the demonstration of the critical roles of the cerebellum in cognitive, emotional, and motor processes. Increased information capacity multiple cell type and feedback circuit cerebellar cortex complex neural networks have been found in studies on the cerebellar cortex (Yang et al., 2024). Further, studies on correlations between neurodegenerative disease like Parkinson's and Alzheimer's and cerebellar atrophy have provided greater awareness of cerebellar involvement in disease pathophysiology and treatment options (Sivalingam, 2024). Cerebellar function has been better mapped with newer methods like cerebellum-specific activation-likelihood estimation (C-SALE), which has delineated the cerebellum's role in cognitive and affective processing (Magielse et al., 2024). Additionally, research on fetal alcohol spectrum disorders has shown the cerebellum's sensitivity to prenatal alcohol exposure and the necessity for targeted diagnostic and therapeutic interventions (Leung et al., 2024, Holloway et al., 2023). Together, these findings shed light on the many functions of the cerebellum in health and disease and imply that much more study of how it interacts with the cerebral cortex is necessary. Future medical research has the potential to make a major difference in clinical practice across many different specialties. Enhancement of communication among medical professionals and patients with life-limiting illnesses is vital, focusing on advance care planning, shared decision-making, and culture responsiveness to improve quality of care and outcomes for patients (Ghanem et al., 2024). Use of artificial intelligence (AI) in personalized medicine is another one that is imperative. Once ethical and legal problems are solved, AI can boost the accuracy of diagnoses, tailor treatment protocols, and enhance patient-provider communication (Subramaniam, 2023, Fatima et al., 2024, Schirizzi et al., 2023). Investigation of immunotherapy and angiogenesis inhibitors for tumors like biliary tract cancers continues in oncology; additional clinical trials will be necessary to enhance such approaches and overcome immunosuppressive tumor microenvironments (Kowalski et al., 2022). Apart from bridging gaps in diagnosis, to enable effective therapies to be rolled out for enhancing care globally, the instruments such as the Implementation Planning Assessment Tool are also being developed to bridge gaps between clinical trial results and practice (Timpel and Harst, 2019). Another emerging area needing rigorous technology assessments, high-quality research, and strong implementation strategies is telemedicine, especially in chronic disease management (Timpel and Harst, 2020). This is anticipated to revolutionize our knowledge about and management of serious mental disorders like schizophrenia. As improvements in patient care and addressing the ethical and effective employment of new technology are brought forward by these advances, so too does it bring into focus the relevance of innovation and interdisciplinary cooperation in the healthcare system (Esteban et al., 2025). There are still a lot of unanswered questions about the particular processes of cerebro-cerebellar interactions, despite the mounting evidence of cerebellar contribution to cognition (Zhou et al., 2024). How particular regions of the cerebellum are engaged in specific cognitive processes, such as working memory, executive control, and language processing, is an area of interest. The precise mechanisms and pathways are still not known despite the suggestion from neuroimaging studies of bidirectional communication between the cerebellum and the higher-order brain regions like the prefrontal cortex (Zhang et al., 2023). Furthermore, it is not known how cerebellar neuroplasticity regulates learning and adaptation within the cell and circuits. Whether the cerebellar neuroplasticity has an impact on cerebral plasticity and vice versa needs to be investigated further. Another open question is the function of the cerebellum in neurodevelopmental and psychiatric disease. While diseases such as attention deficit hyperactivity disorder (ADHD) (Reduced cerebellar volume, disrupted fronto-cerebellar loops), schizophrenia (Altered Crus I/II connectivity with prefrontal cortex), and autism spectrum disorder (ASD) (Vermis hypoplasia, abnormal Purkinje cell counts.) have been associated with cerebellar impairment, the pathophysiology is unknown (Rudolph et al., 2023). The development of targeted treatments hinges on an understanding of whether cerebellar impairment is an etiological cause or a disease consequence. The intricacy of cerebro-cerebellar connections is possible to explain with the aid of advances in neuroscience technology. Decomposing the function of certain cerebellar circuits in cognition as well as their impact on cortical activity can become simplified through the use of optogenetics, which makes use of light-sensitive proteins in an effort to manage the brain activity extremely precisely (Jobson et al., 2024). Diffusion tensor imaging (DTI) and functional magnetic resonance imaging (fMRI) are refining mapping of cerebellar-cerebral connections in health and disease. Large-scale brain circuit mapping, or connectomics, is another emerging field that may be able to reveal the cerebro-cerebellar network. Connectomics is able to illustrate how disruption of cerebellar-cortical circuits leads to cognitive and motor disturbances using high-resolution imaging and computer modeling. Transcranial magnetic stimulation (TMS) and transcranial direct current stimulation (tDCS) are both non-invasive brain stimulation methods that are being researched for their potential to improve cerebellar function and reorganize cerebro-cerebellar connections (Hua et al., 2022). Drawing from what is known from research about neuroplasticity, translational research should be aimed at implementing individualized treatments of brain and cerebellar disease. For such disorders as ASD, ADHD, and schizophrenia, personalized neuromodulation approaches such as DBS or neurofeedback training are likely to be created to re-create functional connections (Stoodley, 2016). Research has proven that the cerebellum modifies structurally based on brain trauma and neurodegeneration, and has made stroke rehabilitation methods and methods for neurodegenerative disease another compelling treatment option (Brady et al., 2019). Cognitive as well as physical therapies with enhancements in cerebellar neuroplasticity have the potential to benefit stroke victims as well as Alzheimer’s individuals. Furthermore, the pharmacological research into drugs augmenting cerebellar plasticity can provide emerging therapies for cerebellar and motor disease. These strategies represent a measure of the importance placed on translational research in providing the bridge from clinical application through scientific innovation aimed at enhancing the outcome for cerebral and cerebellar diseases (Manto et al., 2012).

## 3 Conclusion

This review synthesizes evidence establishing the cerebro-cerebellar system as a critical integrator of motor, cognitive, and emotional processes. We have outlined a framework that connects the cerebellum's functional topography and its predictive computational principles to specific pathophysiological mechanisms in neuropsychiatric and neurodegenerative disorders. The cerebellum's role extends far beyond motor coordination, significantly contributing to conditions like schizophrenia, ASD, and ataxia through disrupted cerebro-cerebellar loops. While this synthesis advances a more integrated view, several limitations in the current body of literature persist, including a reliance on cross-sectional studies, heterogeneous patient populations, and an underrepresentation of molecular-level mechanisms in human studies.

## CRediT authorship contribution statement

**Munna Kumar Podder:** Writing – review & editing, Resources. **Rubait Hasan:** Validation, Resources. **Mst. Mohona Khatun:** Writing – original draft, Methodology, Investigation, Formal analysis, Data curation. **Mohammad Shahangir Biswas:** Writing – review & editing, Writing – original draft, Supervision, Conceptualization. **Mst. Ayesha Siddika:** Writing – review & editing, Visualization.

## Consent for publication

Not applicable.

## Ethics approval and consent to participate

Not applicable.

## Funding

None.

## Declaration of Competing Interest

The authors declare that they have no known competing financial interests or personal relationships that could have appeared to influence the work reported in this paper

## Data Availability

Not applicable
